# Metabolite Identification Data in Drug Discovery, Part 2: Site-of-Metabolism Annotation, Analysis, and Exploration for Machine Learning

**DOI:** 10.1021/acs.molpharmaceut.5c00740

**Published:** 2025-10-21

**Authors:** Ya Chen, Susanne Winiwarter, Roxane Axel Jacob, Marie Ahlqvist, Angelica Mazzolari, Filip Miljković, Johannes Kirchmair

**Affiliations:** 1 Department of Pharmaceutical Sciences, Division of Pharmaceutical Chemistry, Faculty of Life Sciences, 27258 University of Vienna, Josef-Holaubek-Platz 2, 1090 Vienna, Austria; 2 Medicinal Chemistry, Research and Early Development, Cardiovascular, Renal and Metabolism (CVRM), BioPharmaceuticals R&D, 468087 AstraZeneca, Pepparedsleden 1, 43183 Mölndal, Sweden; 3 Drug Metabolism and Pharmacokinetics, Research and Early Development, Cardiovascular, Renal and Metabolism (CVRM), BioPharmaceuticals R&D, 468087 AstraZeneca, Pepparedsleden 1, 43183 Mölndal, Sweden; 4 Vienna Doctoral School of Pharmaceutical, Nutritional and Sport Sciences (PhaNuSpo), 27258 University of Vienna, 1090 Vienna, Austria; 5 Dipartimento di Scienze Farmaceutiche, Università degli Studi di Milano, 20133 Milano, Italy

**Keywords:** xenobiotic metabolism, drug metabolism, sites of metabolism (SoMs), data sets, data annotation, data analysis

## Abstract

The ability to pinpoint and predict sites of metabolism (SoMs) is essential for designing and optimizing effective and safe bioactive small molecules. However, the number of molecules with annotated SoMs is limited, hindering the advancement of data-driven methods such as machine learning for metabolism prediction. Here, we provide a comprehensive characterization of SoM data obtained from the readouts of a human hepatocyte assay conducted at AstraZeneca Gothenburg. We explore a new strategy for SoM annotation that accounts for uncertainty in the experimental data, and we relate our findings to the most comprehensive SoM data collection available to date. Our study includes entropy analysis of SoM annotations, accompanied by representative examples that highlight the complexities of interpreting and working with metabolism data. Furthermore, we demonstrate the impact and value of the new metabolism data on SoM prediction. Importantly, a substantial portion of the data generated and analyzed as part of this work is made publicly available.

## Graphical abstract

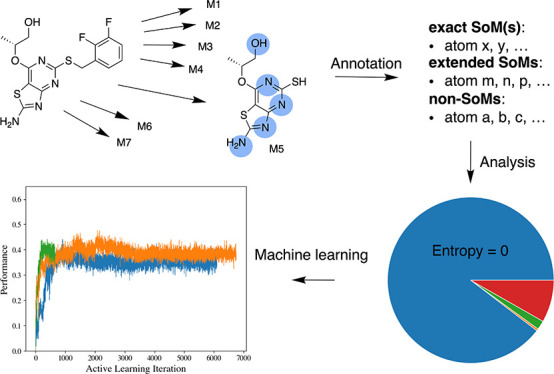

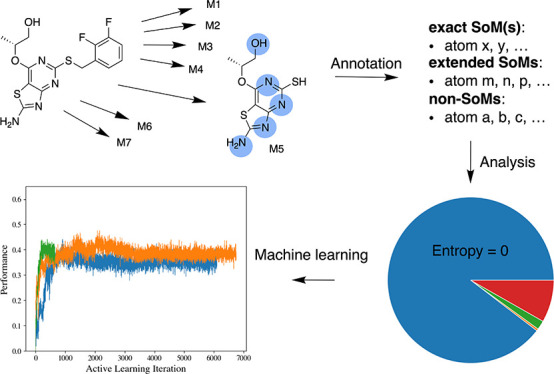

## Introduction

Biotransformation can decisively impact the efficacy and safety of small organic molecules, including drugs, cosmetics, and agrochemicals. Many powerful experimental approaches are available today to predict and determine these effects. However, they remain resource and time intensive. Therefore, computational methods for predicting xenobiotic metabolism have gained considerable interest.^1–3^ Some of these methods have the potential to enable interactive and fully automated compound optimization.

The atom positions in a molecule where metabolic transformations occur are typically referred to as sites of metabolism (SoMs). The prediction of SoMs in the context of small molecules is particularly relevant to medicinal chemists. Once identified, researchers can use SoM information to effectively design and optimize a compound’s metabolic properties while maintaining its desired bioactivity and physicochemical characteristics. Similarly, some metabolite structure predictors, such as GLORYx,^4^ MetaSite,^5^ Meteor,^6–8^ and XenoNet,^9,10^ utilize predicted SoMs to filter and rank predicted metabolites, thus aiding, among other analyses, in the early assessment of potential safety liabilities.

Several SoM predictors are available today, most of which incorporate machine learning components.^11–21^ Among these, FAME 3 ^11^ is one of the few freely accessible SoM predictors covering both phase 1 and phase 2 metabolism. Phase 1 metabolism involves functionalization reactions such as oxidation, reduction, and hydrolysis to introduce or expose functional groups. The primary enzymes involved in phase 1 metabolism are cytochrome P450 enzymes. Phase 2 metabolism involves conjugation reactions, which lead to the attachment of polar moieties, such as glucuronic acid, sulfate, or glutathione, to increase solubility and promote excretion. These reactions are typically catalyzed by transferases, and often further transform functional groups introduced during phase 1 metabolism. For holdout data, FAME 3 identified at least one experimentally observed SoM among the top 2 positions of rank-ordered atom lists for over 80% of the test compounds.^11,22^

Most noncommercial SoM predictors are trained on the Zaretzki data set.^18^ This data set comprises 680 drugs and drug-like molecules annotated with their experimentally determined SoMs resulting from cytochrome P450-mediated biotransformations. With 2000 annotated drugs and drug-like molecules, the MetaQSAR database^23^ exceeds the size of the Zaretzki data set. More importantly, the MetaQSAR database covers the full range of phase 1 and 2 enzymes involved in xenobiotic metabolism.^24^

A characteristic feature and important limitation of most biotransformation data sets is their high heterogeneity. They typically comprise data from various literature sources, collected using distinct technologies on distinct biological materials and systems (purified proteins, cells or cell homogenates, animal models, etc.). It is common for these data sets to cover multiple species, particularly mammalian ones.

The location of a SoM is primarily determined by its local atom environment. These atom environments show redundancies across the chemical space,^11^ for which reason SoM predictors typically have a broader applicability domain (in terms of molecular space) than predictors of global molecular properties. However, a broader molecular context surrounding an atom can also be decisive for its metabolic stability.^15^ For example, bulky substituents can enhance metabolic stability by steric hindrance.

The available SoM data covers only a fraction of the molecule space relevant to drug discovery and related domains. For further advancement of SoM predictors, high-quality SoM data covering distinct atom environments, as well as rare and complex biotransformations, will be essential. Such information can be derived from metabolite identification (MetID) schemes. Recently, methods for automated SoM annotation from biotransformation data have become available.^25^ However, these require machine-readable and properly preprocessed input.

Recently, we reported on the generation, characterization, and publication of metabolite scheme data.^26,27^ The published data set includes the structures of phase 1 and phase 2 metabolites observed in human hepatocyte incubation experiments conducted at AstraZeneca, derived using ultraperformance liquid chromatography–mass spectrometry (UPLC-MS).

In continuation of this work, we report here on the annotation of 217 parent compounds with SoM labels (Figure 1). To account for the uncertainty in the experimental data, we explored two distinct definitions for SoMs:

1. Exact SoMs: These include all atoms with direct experimental evidence of metabolic lability, as the exact metabolic product has been identified.
2. Extended SoMs: These include all exact SoMs plus all regions in a molecule (i.e., atom groups or SoM groups) where metabolic lability was detected but could not be pinpointed to the specific labile atom.

**Figure 1:**
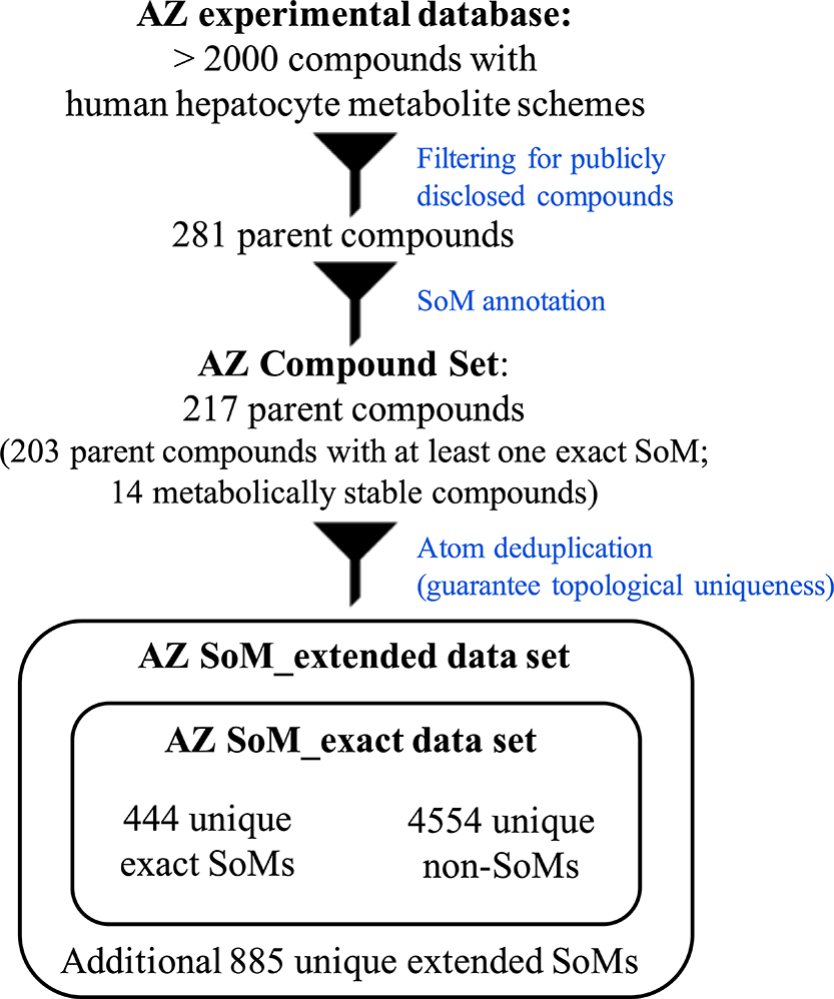
Data set compilation and composition. From over 2000 compounds with human hepatocyte metabolite schemes stored in the metabolism database of AstraZeneca (AZ) Gothenburg, we annotated the SoMs of 217 publicly disclosed parent compounds with valid human hepatocyte assay data records.

Our SoM annotation strategy is illustrated in Figure 2 and explained in full detail in the Methods section.

**Figure 2:**
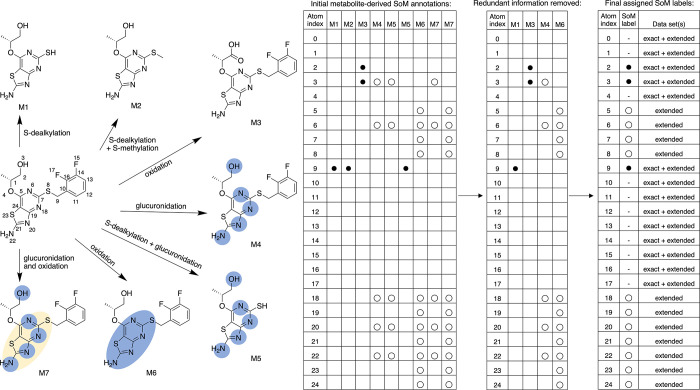
Illustration of the SoM annotation logic applied to ultraperformance liquid chromatography–mass spectrometry (UPLC-MS) data. In cases where the available UPLC-MS data enables the definition of a metabolite structure (M1, M2, and M3), the atoms involved in forming this specific metabolite are labeled as exact SoMs, represented as solid circles in the tabular overview. In cases where a metabolite structure cannot be derived from the UPLC-MS data (M4, M5, M6, and M7), all atoms potentially involved in the observed biotransformations are labeled as extended SoMs in a grouped manner. In the tabular overview, these SoM groups are represented as hollow circles; in the structure diagrams, they are indicated as blue and yellow areas (the use of two colors indicates that, in this example, two reaction types are observed). The fact that some of these areas span two or more atoms reflects the uncertainty in the measured UPLC-MS data regarding the precise location of SoMs. In cases where distinct biotransformations result in the same metabolite structure (M5 and M7), the SoM annotations are derived individually and then merged onto one molecular structure, observing the following rules. Rule 1: An atom is labeled as an exact SoM if experimental evidence is available for the presence of at least one metabolite related to this SoM. Rule 2: An atom is labeled as a non-SoM (represented by a hyphen in the tabular overview) if it was never observed to be involved in a biotransformation. Rule 3: All remaining atoms (i.e., all atoms for which UPLC-MS data indicates that they are potentially involved in biotransformation, but the exact SoM cannot be pinpointed) are labeled as extended SoMs (hollow circles).

We provide a comprehensive characterization of this new data and compare it to the MetaQSAR data set. The characterization includes entropy analysis of SoM labels and a detailed examination of representative cases, highlighting the complexities involved in interpreting metabolism data and developing predictive models. Lastly, we explore the value of the new data for developing machine learning models for SoM prediction.

## Methods

### Compilation of the AstraZeneca SoM Data Sets

A total of 281 data records of publicly disclosed parent compounds were sourced from the MetID database of AstraZeneca Gothenburg. Each of these compounds is linked to MetID schemes originating from human hepatocyte incubation experiments analyzed using UPLC-MS. The experimental procedure is described in ref 26.

For a subset of 217 constitutionally unique parent compounds (“AZ Compound Set”), we were able to conduct SoM annotation according to the scheme exemplified in Figure 2 (note that 14 of the 217 compounds were determined to be metabolically stable in the experiments, hence not containing SoMs). For the example provided in Figure 2, the available UPLC-MS data confirms the presence of metabolite M1 and, hence, a SoM at atom position 9 of the compound, related to an S-dealkylation reaction. Similarly, for metabolite M2, position 9 is reconfirmed as a SoM (S-methylation reaction following the S-dealkylation reaction). Positions 2 and 3 are confirmed by metabolite M3 (alcohol oxidized to carboxylic acid). However, there is uncertainty regarding the exact locations of the SoMs leading to M4, M5, M6, and M7. For M4, the UPLC-MS data simultaneously indicate a single glucuronidation reaction (based on the detected molecular mass) occurring at either any nitrogen atom of the parent compound (*N*-glucuronidation at position 6, 18, 20, or 22) or its terminal alcohol moiety (O-glucuronidation at position 3).

To address the uncertainty regarding the locations of SoMs, the MetID schemes were translated into two data sets of SoM atoms: AZ SoM_exact includes all atoms labeled as SoMs that can be accurately identified based on the available experimental data (positions 2, 3, and 9 of the parent compound shown in Figure 2). AZ SoM_extended adds groups of atoms labeled as SoMs (SoM groups) for which the precise locations cannot be identified based on the experimental data (atom positions 5 to 8 and 18 to 24 of the parent compound shown in Figure 2), as compared to the AZ SoM_exact set. Only atoms that were never identified as SoMs were labeled as non-SoMs, and these atoms are included in both the AZ SoM_exact and SoM_extended data sets. Thus, the AZ SoM_exact data set is a subset of the AZ SoM_extended data set.

### Calculation of Representations for Molecules and Atoms

The molecular structures of compounds in the AZ Compound Set, the SoM database MetaQSAR, and the Approved Drugs subset of DrugBank^28,29^ (employed as a reference data set to represent the chemical space of drugs) were prepared according to the procedure described previously.^22^ This involved standardizing the molecular structures and removing all salt components utilizing the ChEMBL Structure Pipeline.^30,31^

Of the 217 parent compounds in the AZ Compound Set, (i) all are parseable with RDKit^32^ without errors, (ii) none contain element types other than C, N, S, O, H, F, Cl, Br, I, P, B, and Si, (iii) all have a molecular weight between 100 and 1000 Da, and (iv) none are duplicates (checked using InChI, disregarding stereochemical information).

Of the 2618 compounds in the Approved Drugs data set, 2179 passed all aforementioned preprocessing steps. The removed compounds include 224 that violate the element filter, 142 that fall outside the molecular weight range, and 73 duplicate molecules (based on InChIs, excluding stereochemical information).

Of the 2314 compounds in the MetaQSAR database, 1926 passed all preprocessing steps. A total of 388 compounds were removed, including 21 compounds that violate the element filter, 71 that are outside the allowed molecular weight range (100 to 1000 Da), and 296 duplicates (based on InChIs, excluding stereochemical information).

Carefully selected sets of descriptors were calculated with RDKit and CDPKit^33^ and employed to characterize the molecular properties and atom environments of the compounds represented in the individual data sets (Table 1).

**Table 1:** Overview of Descriptors and Representations Employed in This Study

structural representation level	task	descriptors, representations	explanation	method(s)
molecule	characterization of the chemical space covered by a set of molecules	15 physicochemical properties^a^ calculated with RDKit	these descriptors capture the fundamental physicochemical and structural properties relevant to small-molecule research	UMAP
molecule	quantification of pairwise molecular similarity	Morgan fingerprints calculated with RDKit (radius 2, 2048 bits)	these fingerprints capture substructural patterns and are well-suited for binary vector-based molecular similarity analysis	Tanimoto coefficient
atom	characterization of the atom environments covered by a set of molecules	FAME descriptors calculated with CDPKit (bond path length of 5)	high-content descriptors capturing element types, hybridization states, and (further) electronic and topological features	UMAP
atom	generation of machine learning models for SoM prediction			random forest classifier, active learning
atom	quantification of pairwise atom similarity	rooted Morgan fingerprints (radius 1 to 5, 2048 bits) calculated with RDKit^b^	these fingerprints describe the local environment surrounding a central atom	Tanimoto coefficient
atom	identification and counting of unique atom environments			Bitstring identity check

a Number of nitrogen atoms, number of oxygen atoms, number of chiral centers, molecular weight, number of heavy atoms, number of hydrogen bond acceptors, number of hydrogen bond donors, logP, topological polar surface area, number of aromatic atoms, number of rings, fraction of Csp3 atoms, number of sulfur atoms, number of halogen atoms, and molar refractivity.

b Calculated with RDKit’s GetMorganFingerprintAsBitVect function with the central atom specified, ensuring that only bits related to the root atom are included.

**Table 2:** Active Learning Settings Explored in This Work

setup	first model is built on	active learning set	test set	no. of atoms picked during each iteration
1	full MetaQSAR training set	a. AZ SoM_exact data set	MetaQSAR test set (identical to the test set employed in ref 22)	1
		b. AZ SoM_extended data set		
2	a randomly sampled SoM and non-SoM from the active learning set	a. MetaQSAR training set	MetaQSAR test set	5
		b. AZ SoM_exact data set		
		c. AZ SoM_extended data set		
		d. MetaQSAR training set and AZ SoM_exact data set		
		e. MetaQSAR training set and AZ SoM_extended data set		
3	a randomly sampled SoM and non-SoM from the active learning set	a. MetaQSAR training set	AZ SoM_exact random-split test set (atoms from randomly selected 67 molecules in the AZ SoM exact data set)	5
		b. AZ SoM_exact random-split training set (AZ SoM_exact atoms not in the random-split test set)		
		c. MetaQSAR training set and the AZ SoM_exact random-split training set		
4	a randomly sampled SoM and non-SoM from the active learning set	a. MetaQSAR training set	AZ SoM_exact time-split test set, comprising atoms from 67 molecules with experiments conducted in or after 2013	5
		b. AZ SoM_exact time-split training set, consisting of atoms from molecules with experimental data collected before 2013		
		c. MetaQSAR training set and AZ SoM_exact time-split training set		

Uniform Manifold Approximation and Projection (UMAP)^34^ was performed with umap-learn^35^ using default settings.

### Statistical Analysis of SoM Annotations for Unique Atom Environments

For each unique atom environment (determined as defined in Table 1), all SoM labels were collected to calculate the Shannon entropy and confidence scores. Shannon entropy quantifies the uncertainty in the SoM labeling of each atom environment, where each label is either SoM or non-SoM. The occurrences of each label were counted, and their probabilities were calculated by dividing these counts by the total number of occurrences. Using these probabilities, the Shannon entropy was calculated using eq 1:

$$entropy=-p_{1}\log_{2}p_{1}-p_{0}\log_{2}p_{0}$$ 1

where *p*_1_ and *p*_0_ are the probabilities of SoM and non-SoM labels, respectively. The entropy values range from 0 to 1. An entropy of 0 indicates all labels are the same, while an entropy of 1 indicates maximum uncertainty (labels are evenly split between SoM and non-SoM).

Since every atom environment has a different total number of occurrences, a confidence score was calculated using eq 2:

$$confidence\;score=1-\frac{1}{\sqrt{total\;occurrence}+1}$$ 2

The confidence values range from 0 to 1, with values approaching 1 for higher occurrence, indicating higher reliability in the entropy measurement.

### Machine Learning and Active Learning

The SoM predictor FAME 3 is an extra trees classifier generated with scikit-learn,^36^ which uses atom descriptors from CDK.^37^ In this work, we use the reimplementation of FAME 3 described in ref 22. The reimplementation replaces the extra trees classifier with a random forest classifier and CDK with CDPKit. We confirmed that the predictions produced by the original model and the reimplementation are comparable.^22^

For active learning, four setups using various combinations of active learning set and test set were explored (Table 2). Following the active learning approach described in ref 22, models are built through iterative cycles of model training. During each iteration, the model picks one or five most informative atoms from the active learning set to add to the training set, and the new model is evaluated on the test set (which remains untouched during the iterative model training and testing process). All active learning settings were repeated five times, and our previous study confirmed the stability of performance when selecting the five most uncertain atoms each iteration to build a new model compared with picking one most uncertain atom.

### Evaluation of Model Performance

SoM prediction performance was evaluated at the atom level (indicating how well SoMs are identified and ranked among sets of atoms) and the molecule level (indicating how well predictors perform on individual molecules and sets of molecules).

The atom-level performance of the SoM predictor was assessed using the Matthews correlation coefficient (MCC), the area under the receiver operating characteristic curve (AUC), precision, and recall.

The MCC considers all four elements in the confusion matrix (i.e., the number of true positives (TP), the number of true negatives (TN), the number of false positives (FP), and the number of false negatives (FN)). Thus, it is one of the most informative and robust measures for binary classification evaluation (eq 3).

$$MCC=\frac{TP\times TN-FP\times FN}{\sqrt{\left(TP+FP\right)\left(TP+FN\right)\left(TN+FP\right)\left(TN+FN\right)}}$$ 3

The MCC ranges from −1 to 1, where 1 represents perfect classification performance. It was also used to assess the performance of active learning.

The AUC assesses the model’s capacity to classify atoms as SoMs or non-SoMs. It ranges from 0 to 1, with a value of 1 signifying perfect discrimination. Recall (eq 4) evaluates the ratio of correctly predicted SoMs, while precision (eq 5) measures the proportion of true SoMs among all atoms classified as SoMs.

$$recall=\frac{TP}{TP+FN}$$ 4

$$precision=\frac{TP}{TP+FP}$$ 5

The top-*k* success rate measures the proportion of molecules in which at least one known SoM ranks among the top-*k* atom positions within a molecule, with *k* typically taking values of 1, 2, or 3. This metric is influenced by a molecule’s number of atoms and SoMs, a limitation that the more robust Top-*k* Lift Accuracy addresses. Both metrics are thoroughly discussed in ref 38.

## Results and Discussion

### Molecular Space and Physicochemical Property Analysis

The AZ Compound Set comprises 217 publicly disclosed parent compounds with valid human hepatocyte assay data records from the experimental MetID database generated at the AZ Gothenburg site^26^ (Table 3). The data set covers a subspace of the chemical space defined by the Approved Drugs set and the MetaQSAR data set, as shown in Figure 3.

**Table 3:** Composition of the Processed AstraZeneca Compound Set and the MetaQSAR Data Set

	no. of compounds	no. of heavy atoms	no. of SoMs	average no. of SoMs per molecule
AZ Compound Set	217	6476	exact SoMs: 499	exact SoMs: 2.30
			extended SoMs: 1456^a^	extended SoMs: 6.71
MetaQSAR data set (total)^b^	1926	43,418	4976	2.58
MetaQSAR training set (subset)^b^	1505	33,994	3930	2.61
MetaQSAR test set (subset)^b^	421	9424	1046	2.48

a The set of extended SoMs includes all the exact SoMs.

b Numbers taken from ref 22.

**Figure 3:**
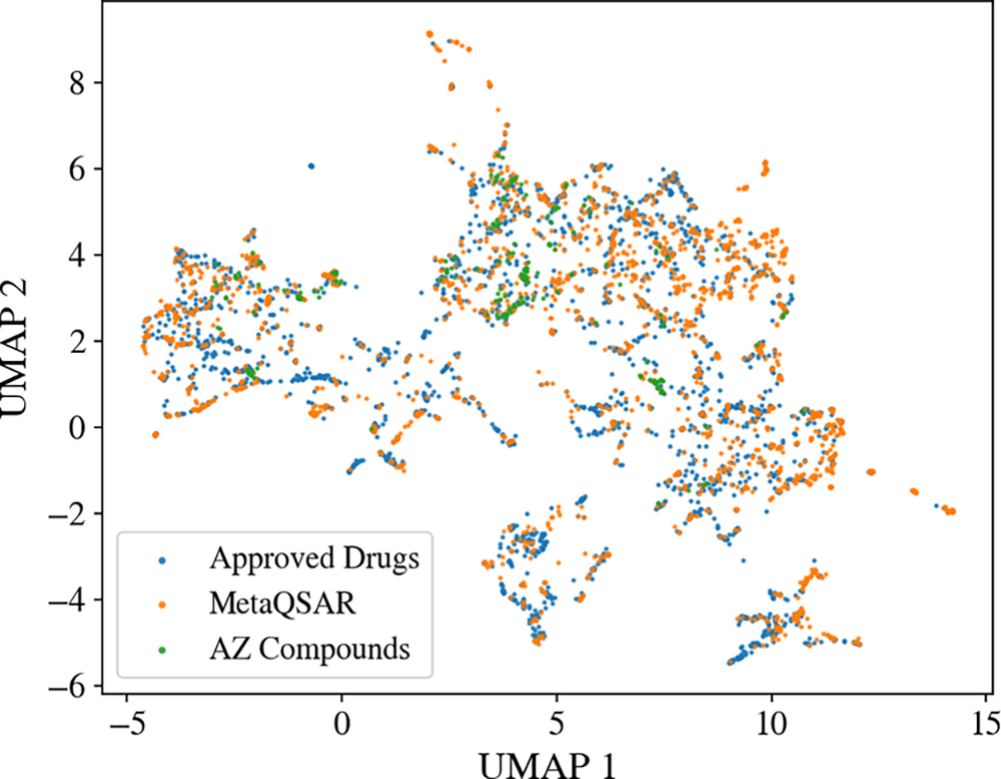
UMAP plot comparing the chemical space covered by the AZ Compound Set, the MetaQSAR data set, and the Approved Drugs subset of DrugBank. The UMAP was generated utilizing 15 physicochemical descriptors (see Table 1 for details).

The overlap between the AZ Compound Set and the MetaQSAR data set consists of only three compounds. Four further compounds in the AZ Compound Set are structurally related to compounds in the MetaQSAR data set (maximum Tanimoto coefficient based on Morgan fingerprints (radius of 2 and 2048 bits) greater than 0.40; see Figure 4A). The overlap between the AZ Compound Set and the Approved Drugs set consists of five compounds; three thereof are structurally related.

**Figure 4:**
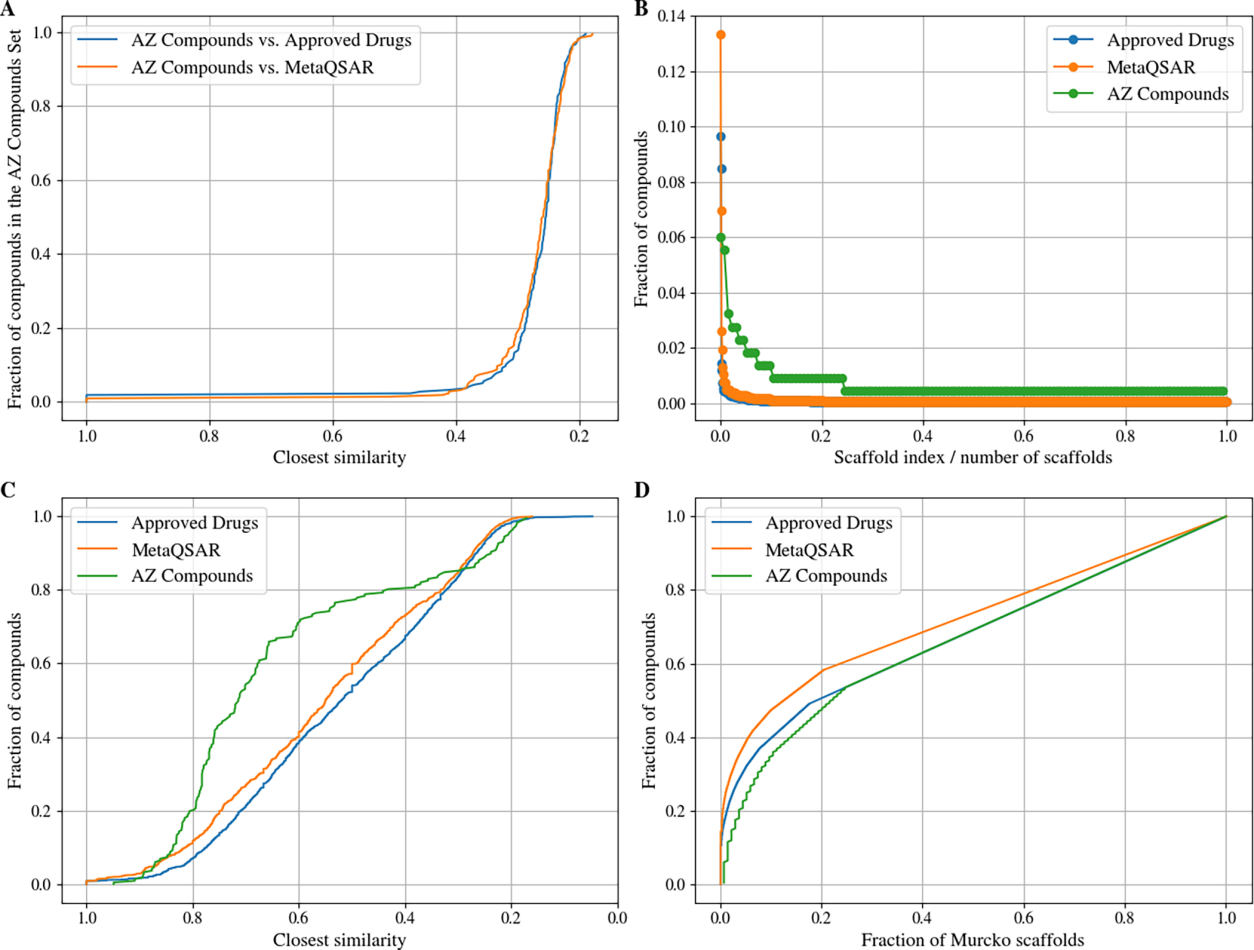
(A) Distribution of Tanimoto coefficients (derived from Morgan fingerprints with a radius of 2 and a length of 2048 bits) for the compounds in the AZ Compound Set and their nearest neighbors in the MetaQSAR and Approved Drugs data sets. (B) Number of compounds represented by each Murcko scaffold within the individual data sets. (C) Distribution of Tanimoto coefficients (identical fingerprint configuration as in (A)) for all compounds and their nearest neighbors within the individual data sets. (D) Cumulative retrieval fraction of the compounds in each data set as a function of the fraction of Murcko scaffolds.

Regarding Murcko scaffolds, the AZ Compound Set represents 134 unique structures, of which 101 account for a single molecule (Figure 4B). Only six Murcko scaffolds are also present in the MetaQSAR data set, four of which are the ubiquitous molecular fragments benzene, pyridine, diphenyl ether, and benzyl phenyl ether. The remaining two scaffolds come from the overlapping compounds (Table S1).

Quantified as a molecular fingerprint-derived Tanimoto coefficient (Figure 4C and Table 1), the molecular diversity within the AZ Compound Set is lower than that in the MetaQSAR and Approved Drugs data sets. However, the plot of the cumulative retrieval fraction of Murcko scaffolds (Figure 4D) indicates that the scaffold count within the AZ Compound Set is particularly high. This discrepancy arises because all compounds in the AZ Compound Set contain at least one ring, whereas 134 compounds (8.49%) in the MetaQSAR data set and 185 compounds (6.96%) in the Approved Drugs data set lack a ring. Compounds without a ring do not contribute to the scaffold count, leading to an underestimation of the molecular diversity of the MetaQSAR and Approved Drugs data sets in scaffold-based analyses. It should also be noted that molecular diversity and data set size can be closely linked.

The fact that the AZ Compound Set covers a subspace within the chemical space of drugs and drug-like compounds is reflected by narrower molecular property distributions (Figure 5). Additionally, the compounds from AZ are characterized by a higher molecular weight and a higher prevalence of nitrogen atoms.

**Figure 5:**
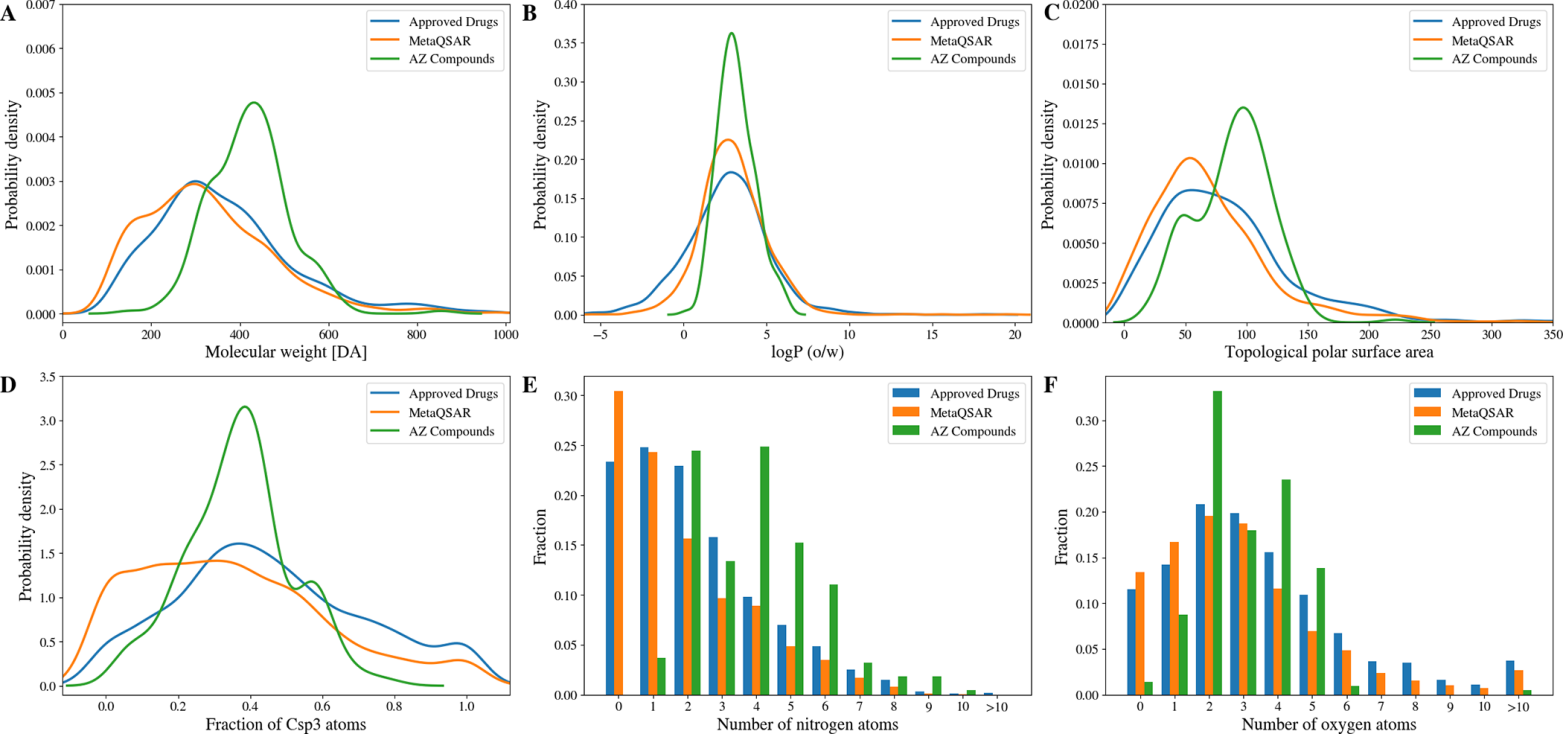
Distribution of key physicochemical properties among the AZ Compound Set, the MetaQSAR, and the Approved Drugs data sets: (A) molecular weight, (B) calculated log P (o/w), (C) topological polar surface area (TPSA), (D) fraction of sp3 hybridized carbon (Csp^3^) atoms, (E) number of nitrogen atoms, and (F) number of oxygen atoms.

### Atom Space Analysis

To understand how the AZ Compound Set compares to the MetaQSAR and Approved Drugs data sets regarding the coverage of atom environments, we conducted a series of data analysis and visualization studies. To enhance the expressiveness of these experiments, we removed duplicate SoM data related to topological identity (for example, an unsubstituted terminal phenyl moiety undergoing a hydroxylation reaction in the meta position will have two topologically identical atoms labeled as SoMs). This processing of the AZ Compound Set resulted in two atom data sets: the AZ SoM_exact data set, consisting of 445 topologically unique exact SoMs and 4548 topologically unique non-SoMs, and the AZ SoM_extended data set, comprising 1335 topologically unique SoMs and 4548 topologically unique non-SoMs (Table 4). Consistent with the definitions outlined in the Introduction and Methods sections (and visualized in Figure 1), the AZ SoM_extended set includes all SoMs from the AZ SoM_exact set.

**Table 4:** Composition of the AZ and the MetaQSAR-Derived Atom Data Sets

atom set	no. topologically unique atoms	no. topologically unique SoMs	no. topologically unique non-SoMs	fraction of topologically unique SoMs
AZ SoM_exact atom set	4993	445	4548	0.09
AZ SoM_extended atom set (superset)	5883	1335	4548	0.23
preprocessed MetaQSAR data set	38,921	4435	34,486	0.11
MetaQSAR training set (subset)	30,509	3503	27,006	0.11
MetaQSAR test set (subset)	8412	932	7480	0.11

For UMAP, we employed FAME descriptors, as these descriptors are particularly rich in atom information relevant to the task (see Table 1 for details). Similar to what we observed for the molecular space, the UMAP plot presented in Figure 6A indicates that the atom space covered by the AZ SoM_extended and MetaQSAR data sets is comparable (the results for the AZ SoM_exact set are reported in Figure S1; the observations and conclusions are the same).

**Figure 6:**
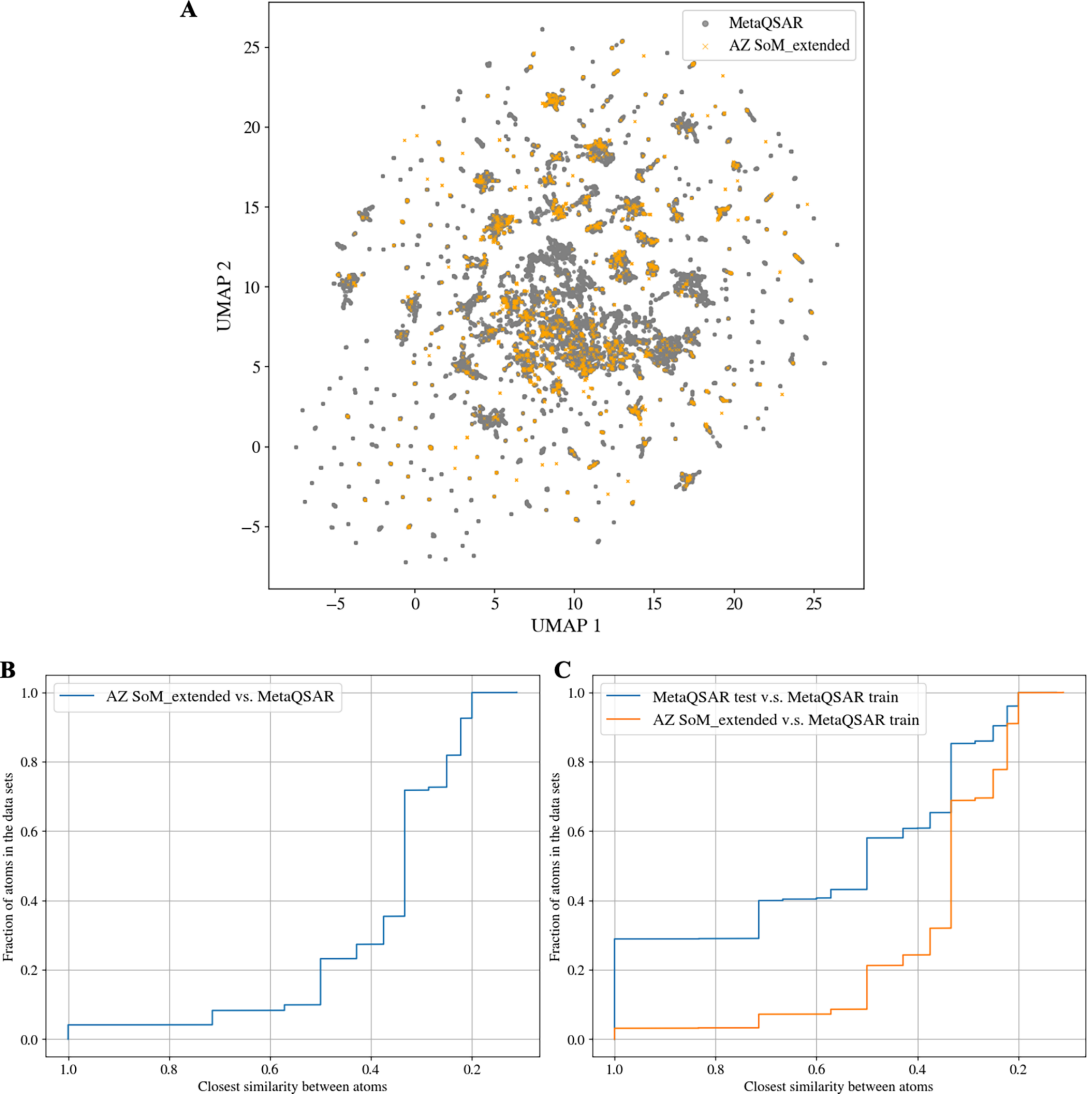
Atom space analysis. (A) UMAP of the atoms comprising the AZ SoM_extended and the MetaQSAR data sets, generated from CDPKit FAME descriptors (with a radius of 5). (B) Distribution of atom environment similarities between the atoms included in the AZ SoM_extended set and their nearest neighbors in the MetaQSAR data set (derived from rooted Morgan fingerprints with a radius of 5 and 2048 bits). (C) Distribution of atom environment similarities between the atoms in the MetaQSAR test set and AZ SoM_extended set, and their respective nearest neighbors in the MetaQSAR training set (derived from rooted Morgan fingerprints with a radius of 5 and 2048 bits). As a subset of the AZ SoM_extended data set, the AZ SoM_exact set shows similar results (Figure S1).

For quantification tasks, we referred to binarized representations of atom environments (i.e., rooted Morgan fingerprints; Table 1) and the Tanimoto coefficient. As shown in Figure 6B, only a small fraction of the atoms in the AZ data sets (roughly 10% of atoms) have atoms with similar atom environments represented in the MetaQSAR data set.

Analyzing the atom environments using rooted Morgan fingerprints of different radii shows varying levels of uniqueness and overlap in atom environments between the AZ SoM_extended and MetaQSAR data sets. When considering only the most proximate atom(s) to the central atom (i.e., fingerprint radius 1), the data sets count 551 and 1951 unique atom environments, respectively. Although the MetaQSAR data set contains more unique atom environments than the AZ SoM_extended data set, the AZ SoM_extended data set is more diverse (representing 10.68 vs 19.95 atoms per unique atom environment).

As larger fingerprint radii are used, the number of unique atom environments increases for each data set (Table 5). In contrast, the number of atoms per unique atom environment (quantifying diversity) declines. In addition, the overlap between the data sets is reduced with larger fingerprint radii: For example, with a fingerprint radius of 1, almost 90% of the atom environments identified in the AZ SoM_extended data set are also found in the MetaQSAR data set, whereas the overlap is reduced to about 45% if the fingerprint radius is 2. With a fingerprint radius of 5 applied (which is identical to the radius applied to the calculation of the FAME descriptors in SoM predictor training and application), this overlap reduces to 4% (details for the AZ SoM_exact set are provided in Table S2; the observed trends are consistent).

**Table 5:** Atom Environments Represented by the AZ SoM_extended and MetaQSAR Data Sets

fingerprint radius	AZ SoM_extended unique atom environments	AZ SoM_extended atom diversity^a^	MetaQSAR unique atom environments	MetaQSAR atom diversity^a^	overlap of unique atom environments	overlap as % of AZ SoM_extended	overlap as % of MetaQSAR
1	551	10.68	1951	19.95	483	87.66	24.76
2	1909	3.08	11,425	3.41	877	45.94	7.68
3	2854	2.06	21,075	1.85	486	17.03	2.31
4	3361	1.75	26,816	1.45	257	7.65	0.96
5	3767	1.56	30,430	1.28	151	4.01	0.50

a Average number of atoms with a unique atom environment.

Interestingly, at a fingerprint radius of 5, 96% of all atom environments in the AZ SoM_exact (and also the SoM_extended data) are not included in the MetaQSAR data set. Likewise, more than 99% of all atom environments in the MetaQSAR are also present in either AZ data sets.

### SoM Annotation Analysis

Analyzing the 217 compounds in the AZ Compound Set, we labeled 597 atom groups as SoMs (an atom group consists of one or more atoms; Figure 1). The 597 atom groups comprise a total of 1456 atoms, 499 of which represent exact SoM locations (Table 3).

The 217 compounds in the AZ Compound Set are, on average, annotated with 2.30 exact SoMs. This value is comparable to the respective rate for the MetaQSAR data set (2.58 SoM atoms per compound). For the extended annotations, the average number of SoMs per molecule increases to 6.71 SoMs. Figure 7 shows the distribution of SoM annotations across the molecules of both the AZ and MetaQSAR data sets. The graph confirms a similar distribution of the number of exact SoMs per molecule for both the AZ Compound Set and the MetaQSAR data set.

**Figure 7:**
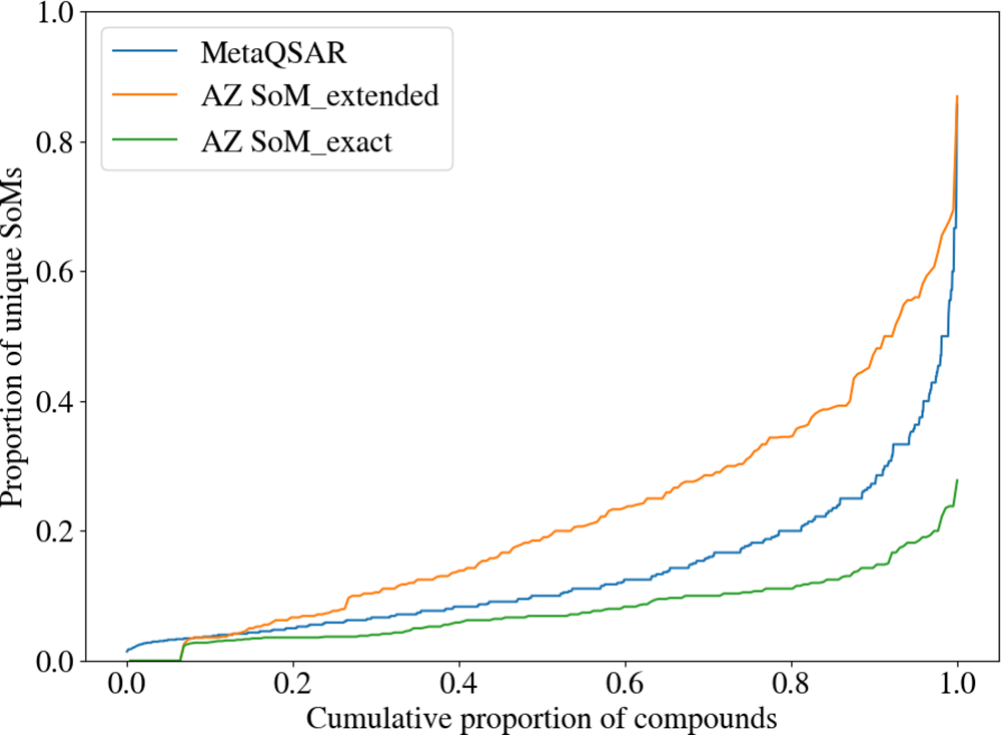
Cumulative distribution of the proportion of SoM atoms within the AZ SoM_exact, the AZ SoM_extended, and the MetaQSAR data sets. The curve progression indicates the presence of compounds with a high proportion of SoMs in both the AZ SoM_extended and MetaQSAR data sets. In the AZ SoM_extended data set, this reflects compounds with large groups of atoms for which the SoM location cannot be pinpointed. In contrast, the peak observed for the MetaQSAR database is mainly related to very small, and sometimes symmetric, molecules (such as valproic acid), where even a low number of SoMs results in a high proportion.

Previous studies of SoM predictors have shown that atomic environments present varying levels of difficulty in SoM prediction.^39^ This is due to differences in the availability and consistency of training data, physicochemical properties of the compounds, and distinct characteristics of the metabolizing enzymes.

Clearly, the metabolic lability of some atom environments is easier to predict than that of others. For example, predicting hydroxy substituents of aromatic rings as SoM (for glucuronidation reactions) is a simpler task than discriminating whether a hydroxylation reaction will occur in the meta or para position of an aromatic ring.

To understand the consistency and heterogeneity of SoM annotations for different atom environments, we calculated Shannon entropy for the SoM labels for each unique atom environment in the AZ data sets. Since increasing fingerprint radii leads to a steep decline in instances representing a specific atom environment (Table 5), we explore radii of 1 and 2 and provide confidence values to account for data density. By prioritizing data robustness over quantity, we focus this analysis on the AZ SoM_exact data set.

Figure 8A illustrates the frequency of unique atom environments in the AZ SoM_exact data set. Using rooted Morgan fingerprints of radius 1, the percentage of unique atom environments represented by at least six samples is around 35%. With a radius of 2, this percentage is reduced to 12%.

**Figure 8:**
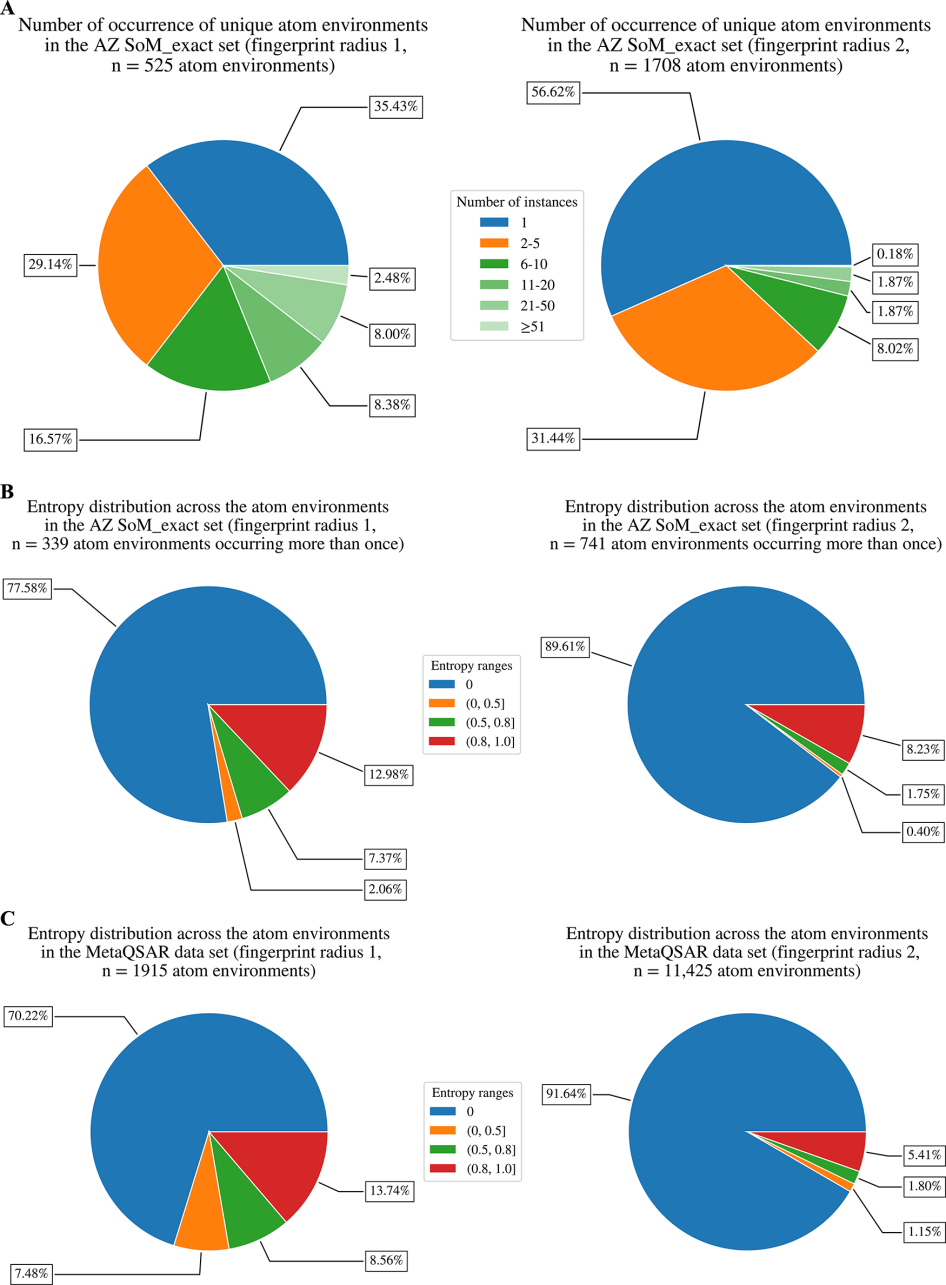
(A) Number of occurrences of unique atom environments based on rooted Morgan fingerprints. (B) Entropy distribution for the atom environments in the AZ SoM_exact data set. (C) Entropy distribution for the atom environments in the MetaQSAR data set.

Entropy is low across the AZ SoM_exact data set (Figure 8B). This speaks for high data quality and homogeneity, partly due to the consistent assay setup at AZ. With a fingerprint radius of 1, for the 339 atom environments occurring more than once, 78% of the unique atom environments are consistently labeled as SoM or non-SoM (i.e., their entropy is 0). With a fingerprint radius of 2, the proportion of consistently labeled atoms increases to 90%.

With a fingerprint radius of 2, only 16 atom environments represented at least 10 times have entropy values greater than 0. Among these, the eight atom environments with the highest entropy values are reported in Table 6. The maximum entropy value was noted for a methyl substituent attached to a nitrogen-containing heterocycle (Table 6, substructure 1). Figure 9 shows structurally related parent compounds containing the substructure in question, specifically, a methyl group attached to a pyrazole. The moiety can undergo demethylation, albeit only to a small extent. This is why demethylation is not observed or reported for some closely related analogs, particularly for less lipophilic compounds (such as the one shown in Figure 9B). For a more detailed discussion of the specific compound series, readers are referred to ref 26.

**Table 6:**
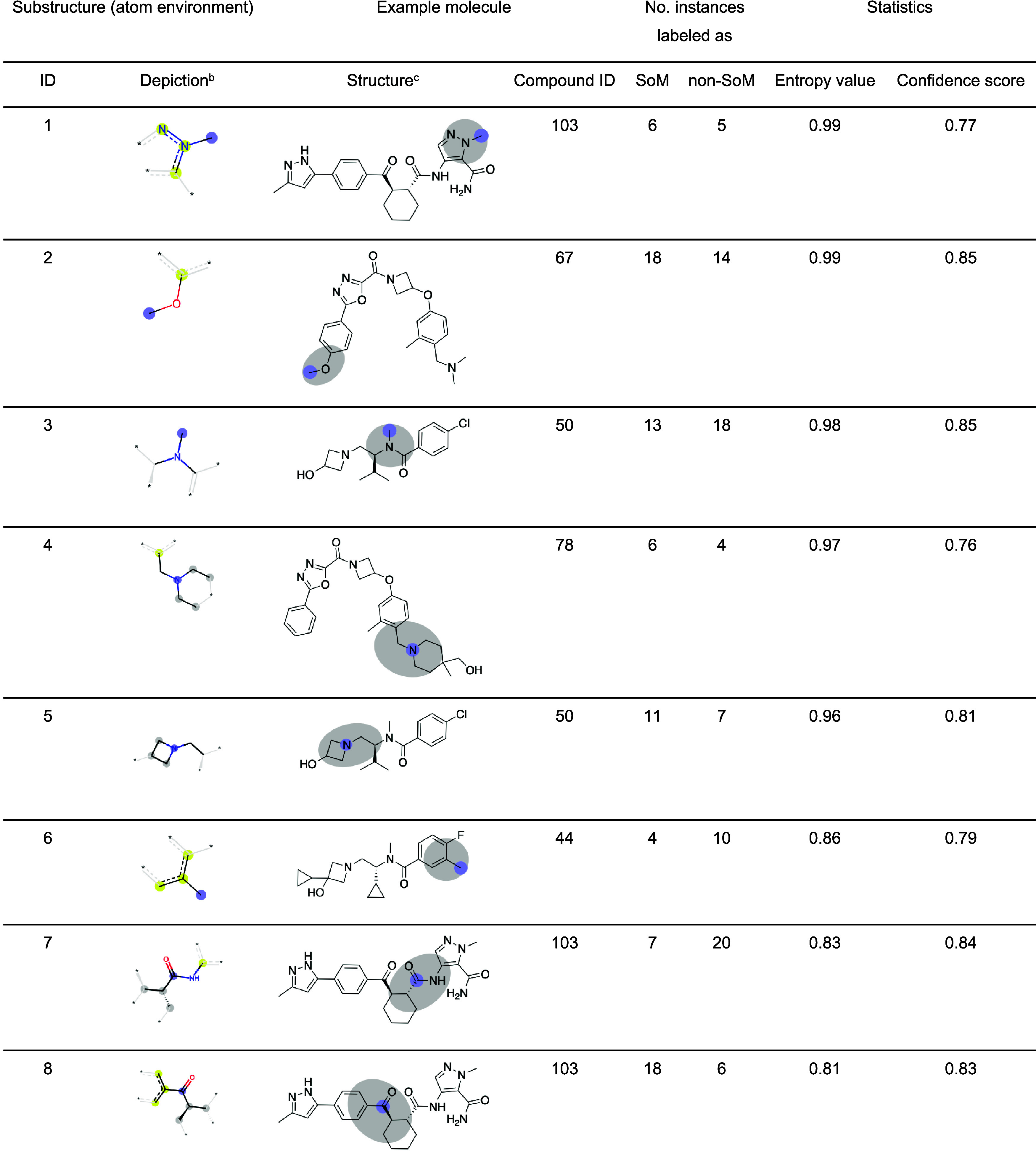
Frequent Atom Environments with High Entropy in SoM Labels^a^

a All atom environments reported in this table are represented by at least 10 instances in the AZ SoM_exact data set. The table is sorted by decreasing entropy values.

b Atom environment derived from RDKit Morgan fingerprints (radius of 2), with the central atom highlighted by a blue circle. All aromatic atoms are highlighted with yellow circles, while gray circles mark aliphatic ring atoms.

c Molecular structures containing the substructure, with the central atom highlighted by a blue circle. Gray circles mark all atoms of the atom environment depictions.

**Figure 9:**
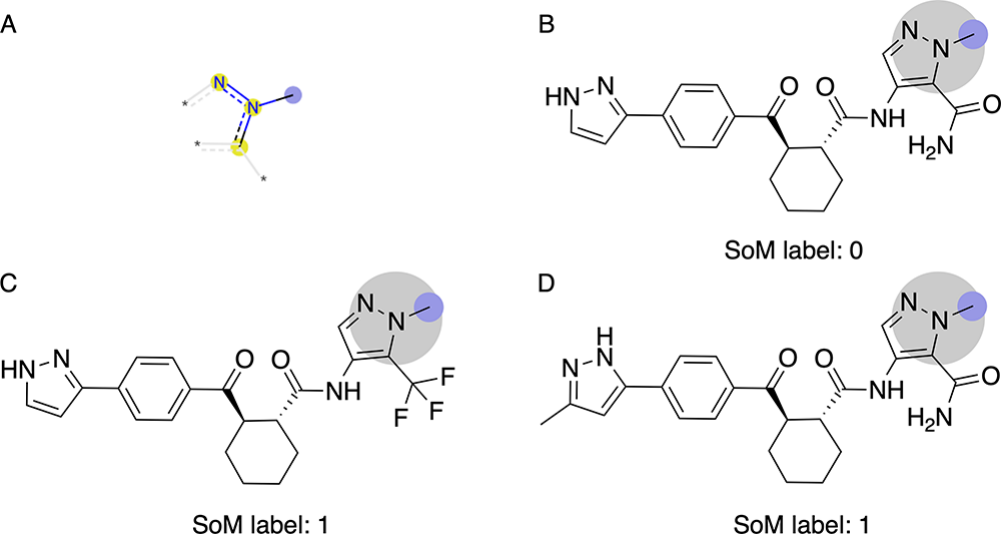
Example of structurally related parent compounds sharing an atom environment where high entropy was observed. (A) Depiction of the relevant substructure, which is a methyl moiety attached to a nitrogen-containing heterocycle (specifically, a pyrazole). (B) Parent compound in which this methyl moiety is reported as metabolically stable. (C, D) Structurally related parent compounds where the methyl moiety is reported as metabolically labile. The likely reason for the discrepancy in the data is low transformation rates, for which the respective metabolites are not observed or reported for some closely related analogues, particularly for less lipophilic compounds.

Discrepancies in SoM annotations are more likely if distinct assay technologies, systems, species, and protocols are employed. This is exemplified in Figure 10: Human hepatocyte data from AZ shows that capromorelin undergoes N-demethylation of the pyrazolidine, O-debenzylation, and hydroxylation of the terminal aminodimethyl moiety. A published in vivo study with rats^40^ reports the N-demethylation and O-debenzylation reactions but not the hydroxylation reaction. Instead, the study reports the hydroxylation of one of the two benzyl moieties.

**Figure 10:**
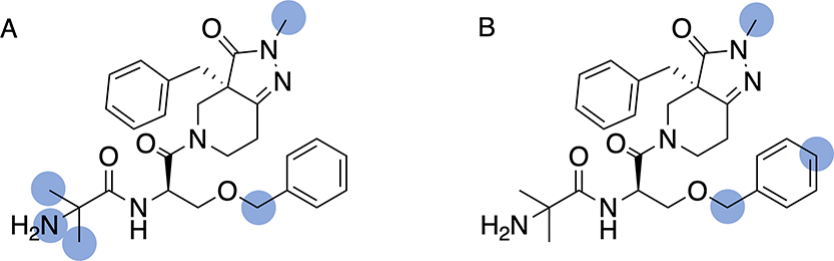
SoMs derived from (A) human hepatocyte data generated at AZ and (B) published data from an in vivo study with rats.^40^

Given that the MetaQSAR data set is compiled from a diverse range of literature covering various experimental assays and even species, the entropy of most SoM labels is surprisingly low. With a fingerprint radius of 2, 79% of the 4564 atom environments occurring more than once in the MetaQSAR data set are consistently labeled as SoM or non-SoM (vs 90% for the AZ_SoM_exact data set; Figure 8B,C).

Importantly, for only 100 atom environments (fingerprint radius 2) among the 797 atom environments (13%) present in both the AZ SoM_exact and MetaQSAR data sets, the entropy increases when merging the two data sets. This indicates that combining both data sets will lead to a larger pool of information while maintaining a low level of aleatoric uncertainty.

### Performance of FAME on the AZ SoM Data Sets

To understand the challenges the AZ SoM data sets present to SoM predictors, we conducted tests using a reimplementation of the SoM predictor FAME 3 (Table 7; see Methods for details). FAME 3 is trained on data from the MetaQSAR database. For performance metrics that require a decision threshold (e.g., the MCC), a value of 0.30 was applied, in line with our previous work.^22^

**Table 7:** Performance of the SoM Predictor FAME 3 Trained on the MetaQSAR Training Set

					standard accuracy^b^			lift accuracy^b^		
test set	MCC^a^	AUC	recall^a^	precision^a^	top-1	top-2	top-3	top-1	top-2	top-3
MetaQSAR test set	0.51	0.89	0.57	0.56	0.73	0.83	0.88	0.67	0.79	0.85
AZ SoM_exact data set	0.37	0.86	0.34	0.51	0.49	0.64	0.71	0.43	0.58	0.65
AZ SoM_extended data set	0.25	0.70	0.18	0.62	0.72	0.85	0.91	0.61	0.76	0.85

a Decision threshold of 0.30.

b Note that these metrics cannot be calculated for the compounds without at least one SoM (which is the case for 14 of the 217 compounds of the AZ Compound Set).

In a MetaQSAR-derived test set (the same test set used in ref 22), the FAME 3 reimplementation achieved an AUC of 0.89 and an MCC of 0.51. While the good AUC value could be maintained for the AZ SoM_exact data set (AUC 0.86), the MCC decreased to 0.37. The observed drop in performance is expected because the similarity of the atom environments represented in the training and test sets is lower (Figure 7C). It is also likely that atom ranking (as indicated by the AUC metric) may be less impacted than atom classification based on a set threshold (MCC metric).

The SoM predictor’s performance declined further on the AZ SoM_extended data set, achieving an AUC of 0.70 and an MCC of 0.25. This additional decline reflects differences in the annotation logic applied to the two data sets (exact SoMs vs metabolically labile regions in a molecule).

A clear difference was observed in the recall, which was much lower for the AZ SoM_extended data set than for the other data sets. Clearly, the reason for this is that only a small subset of atoms among the SoM groups are genuine SoMs. Hence, the observed behavior is one indicator of the validity of the model.

Precision was highest for the AZ SoM_extended data set (Table 7), likely due to the presence of false-positive data points (by definition, only a subset of labeled atoms in the AZ SoM_extended data set are actual SoMs).

SoM predictors are often also evaluated regarding their ability to rank at least one known SoM among the top-*k* positions in a rank-ordered list of atoms. The top-*k* metric quantifies the proportion of such prediction successes across a set of molecules. In line with observations for most performance metrics, the top-*k* values were lower for the AZ SoM_exact set compared to the MetaQSAR test set. For instance, the top-1 values for the MetaQSAR test set and AZ SoM_exact set were 0.73 and 0.49, respectively (Table 7). The top-3 values reached on these data sets were 0.88 and 0.71, respectively.

While easily interpretable, the top-*k* standard accuracy does not account for the statistical difficulty in making a correct prediction. For substrates with a high proportion of SoMs, prediction success will be easier to reach than those with a low proportion of SoMs. This limitation becomes particularly relevant when working with the AZ SoM_extended data set, which has a 2-fold higher rate of SoMs than the AZ SoM_exact and MetaQSAR data sets. The top-*k* Lift accuracy metric accounts for the difficulty posed by test cases at the cost of interpretability.^38^ Overall, the performance trends observed with the top-*k* Lift accuracy metric are consistent with those observed for the top-*k* standard accuracy (Table 7).

As explained previously, the AZ SoM_extended data set is the superset of exactly located SoMs and SoMs located in certain molecule regions. By definition, only a subset of these atoms labeled as potential SoMs will be true SoMs. To understand whether FAME 3 can identify metabolically labile regions in molecules, we assessed its capacity to retrieve at least one atom of such regions. FAME 3 placed at least one atom of a SoM group among the top-1, top-2, and top-3 atom positions for 72, 85, and 91% of the molecules included in the AZ SoM_extended data set (Figure 11). These results confirm the ability of FAME 3 to identify metabolically labile regions in molecules.

**Figure 11:**
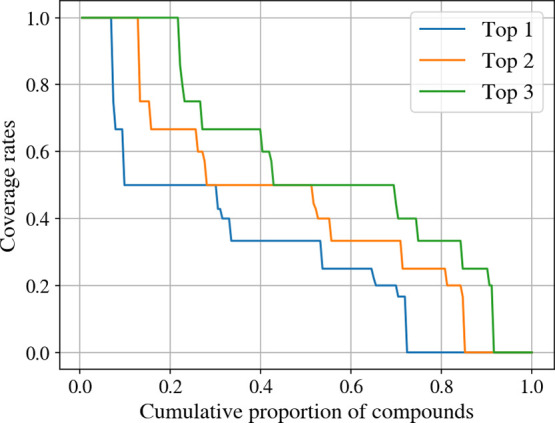
Coverage of SoM groups based on the top-*k* predictions for the AZ SoM_extended data set. A SoM group is regarded as covered if at least one atom from the SoM group is ranked among the top-*k* positions in a molecule.

### AZ SoM Data for Use in SoM Predictor Training

We conducted a series of active learning experiments to investigate whether the AZ SoM data could contribute to building better SoM predictors. In our active learning setup (see the Methods section and ref 22 for details), models are optimized through an iterative cycle of model training. During each iteration, the model selects one or five of the most informative atoms from an atom pool to add to the training set, and the new model is evaluated on the test set (which remains untouched throughout the iterative model training and testing process).

As shown in Figure 12A, starting from the baseline model (i.e., the model trained on the MetaQSAR training set) and expanding its training set with AZ data as part of an active learning process does not yield models that perform better on the MetaQSAR test set. Both the baseline model and the active learning approach that builds on the baseline model yielded MCCs of around 0.51. Likewise, active learning starting from a single atom pair (one SoM and one non-SoM) does not produce models that perform better on the MetaQSAR test set, regardless of the training data used (i.e., individual data sets and combinations thereof; Figure 12B).

**Figure 12:**
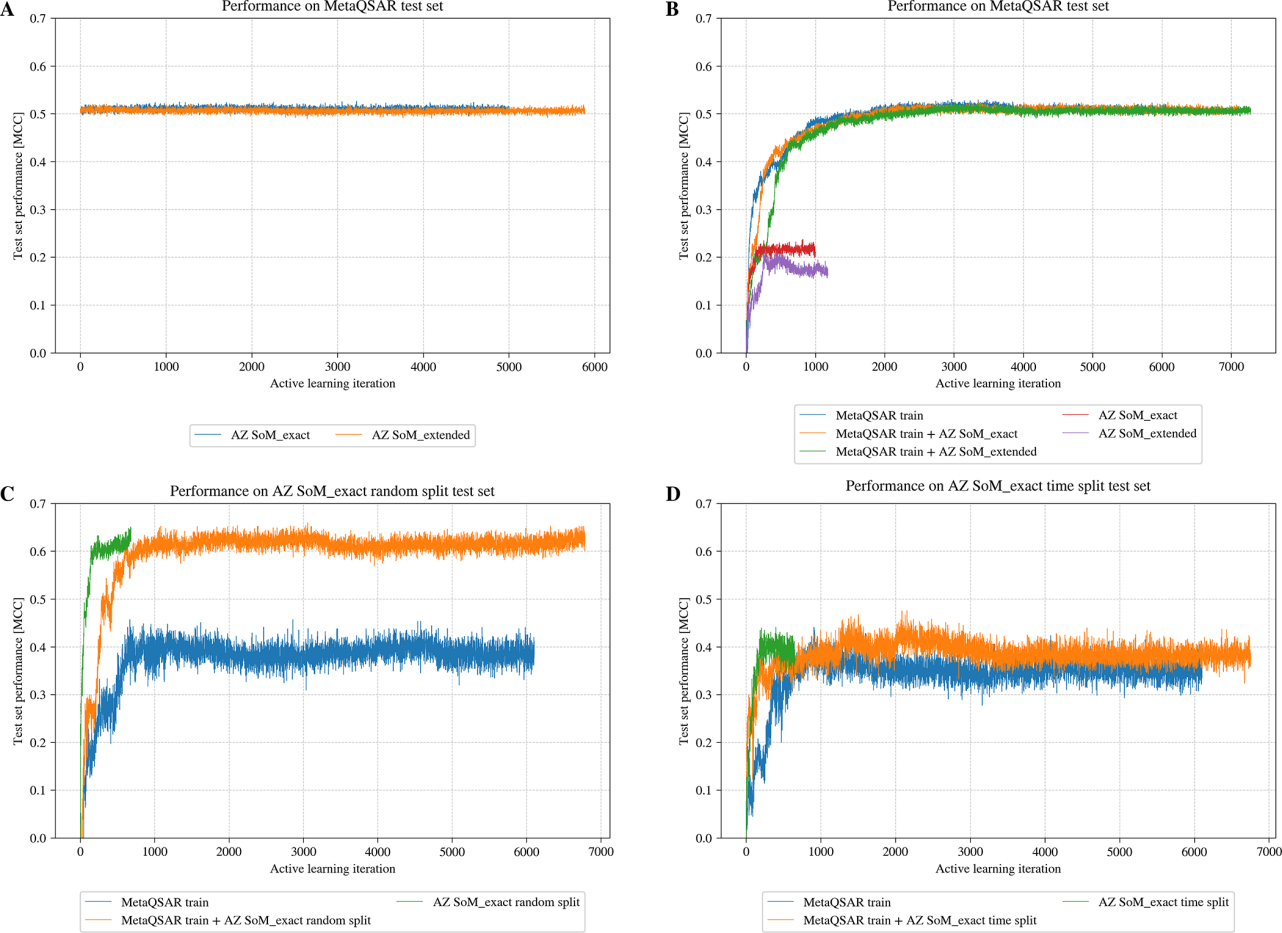
(A) Active learning from the model built on the MetaQSAR training set by adding one atom from one data set for every iteration, and performance measured by MCC on the MetaQSAR test set. (B) Active learning starting from two randomly picked initial atoms and iteratively adding five atoms from one data set or a mixed data set, with performance measured by MCC on the MetaQSAR test set. (C, D) Active learning starting from two randomly picked initial atoms and iteratively adding five atoms from one data set or a mixed data set, with performance measured by MCC on (C) the randomly split test set or (D) the time-split test set from the AZ SoM_exact data set.

Models trained on the AZ data sets performed poorly on the MetaQSAR test set (MCC values around 0.20), but adding the AZ data to the MetaQSAR training set did not degrade the models’ performance, indicating that the AZ data sets do not introduce uncertainty.

Switching the test set to AZ data (random split), active learning yielded well-performing models if, and only if, the training data was, or included, AZ data (without duplicates). This can be attributed to the high similarity between atom environments in the random-split AZ SoM_exact test set and those in the corresponding AZ SoM_exact training set (Figure S2A). As shown in Figure 12C, MCC values of more than 0.60 were obtained in this case. It is also apparent from the figure that only around 1000 AZ data points (30%) are sufficient to yield models that perform well on AZ data. In contrast, models trained solely on the MetaQSAR data and tested on AZ data yield substantially lower performance, with MCC values around 0.40.

To make the AZ test set more challenging, we applied a time-based split: Atoms from molecules with experimental data collected before 2013 were used for training, while atoms from molecules with experiments conducted in or after 2013 were used for testing. The number of compounds in the time-split test set is the same as in the random-split test set. This temporal split reduced the atom similarity between the training and test sets in the AZ SoM_exact data set, bringing it closer to the similarity level observed between the MetaQSAR training set and the AZ SoM_exact test set (Figure S2B). As shown in Figure 12D, models trained on the MetaQSAR training set, the AZ time-split training set, and a combined (mixed) training set all struggled to achieve strong performance, with the best MCC values reaching only around 0.40.

All the active learning settings were repeated five times, and the progression of the models’ performance was found to be stable, consistent with previous observations.^11,22^

## Conclusions

Biotransformation significantly influences the efficacy and safety of small organic molecules. The ability to predict SoMs during the compound design stage can aid medicinal chemists in developing molecules with optimized metabolic properties while preserving desired bioactivities and physicochemical characteristics. The bottleneck in advancing SoM predictors is the coverage of novel atom environments and rare and complex biotransformation events with high-quality experimental data.

This study presents the AZ Compound Set, which comprises 217 compounds meticulously annotated with SoM labels derived from phase 1 and phase 2 metabolite data from a human hepatocyte assay conducted at AstraZeneca Gothenburg. We introduce the concepts of exact and extended SoM annotations, along with group annotations for the extended SoMs, to account for uncertainty in the experimental data.

The AZ Compound Set covers a subspace of the chemical space represented by approved drugs and the MetaQSAR data set. Molecular and atom space analyses show that the AZ Compound Set adds new chemistry, thus making an important contribution to the coverage of the chemical space relevant to drug discovery.

Entropy analyses and detailed examinations of atom environments reveal that the SoM labels for the same local atom environments are largely consistent, indicating the high quality and consistency of the corporate data set. At the same time, individual atom environments showing high entropy values for SoM labels underscore the complexities in interpreting and predicting the metabolic fate of small molecules.

As we demonstrate, the AZ data presents challenges to SoM predictors trained on the MetaQSAR data set due to differences in the experimental technologies and setups used for metabolism analysis, variations in SoM annotation, and disparities in chemical space coverage. Despite these challenges, the FAME model, trained on MetaQSAR data, has demonstrated predictive power and can be applied to distinct chemical spaces. However, the model’s applicability domain must be taken into consideration. As new experimental data on small-molecule metabolism become available, they should continuously be incorporated to update and enhance SoM predictors. Maintaining data consistency and performing compatibility checks will be crucial during this process.

Our results also show that predictive SoM models tailored to compounds of interest can be obtained from small, focused data sets. Active learning has proven to be a particularly powerful strategy when data are scarce.

A subset of 120 compounds with annotated SoMs, derived from proprietary MetID schemes, has been deposited as an open-access resource on the Zenodo platform.^41^ We hope that the scientific community finds the results presented in this work and the accompanying metabolism data useful for further advancement in the field. We also encourage the pharmaceutical industry to double down on its efforts to make data accessible and support community research.

## Supplementary Material



## Data Availability

Of the 281 AZ parent compounds linked to MetID schemes originating from human hepatocyte incubation experiments, the 120 compounds dedicated for MetID data sharing, as per the sister study reporting the data generation and trend analysis of AstraZeneca metabolite identification data for drug discovery,^26^ are publicly available on Zenodo.^41^ The source code of the analyses presented in this work is available from https://github.com/molinfo-vienna/AZ_univie_SoM_data_analysis.

## Notes

Murcko scaffolds in the AZ Compound Set that are also represented in the MetaQSAR data set or the Approved Drugs set (Table S1); UMAP plot of the atoms represented in the AZ SoM_extended and AZ SoM_exact data sets, distribution of atom environment similarities between the atoms included in the AZ SoM_exact set and their nearest neighbors in the MetaQSAR data set, and distribution of atom environment similarities between the atoms included in the MetaQSAR test set and AZ SoM_exact set and their nearest neighbors in the MetaQSAR training set (Figure S1); distribution of atom environment similarities between the atoms included in the AZ SoM_exact test sets and their nearest neighbors in the MetaQSAR training set and AZ SoM_exact training sets, respectively, for both random-split and time-split AZ SoM_exact training set and test set (Figure S2) (PDF)
