# Supplementary material for: Metabolite Identification Data in Drug Discovery, Part 2: Site-of-Metabolism Annotation, Analysis, and Exploration for Machine Learning
Source: Mol Pharm. 2025 Oct 21;22(11):6772–87. doi: 10.1021/acs.molpharmaceut.5c00740 (PMC12587399; doi:10.1021/acs.molpharmaceut.5c00740)
Supplement: Supplementary file 1 [file mp5c00740_si_001.pdf]

Supporting Information

Metabolite Identification Data in Drug Discovery, Part 2: Site-of-Metabolism Annotation, Analysis, and Exploration for Machine Learning

Ya Chen,[1,2] Susanne Winiwarter,[3] Roxane Axel Jacob,[1,4] Marie Ahlqvist,[3] Angelica Mazzolari,[5] Filip Miljković,[2*] and Johannes Kirchmair[1*]

[1] Department of Pharmaceutical Sciences, Division of Pharmaceutical Chemistry, Faculty of Life Sciences, University of Vienna, Josef-Holaubek-Platz 2, 1090 Vienna, Austria

[2] Medicinal Chemistry, Research and Early Development, Cardiovascular, Renal and Metabolism (CVRM), BioPharmaceuticals R&D, AstraZeneca, Pepparedsleden 1, SE-431 83 Mölndal, Sweden

[3] Drug Metabolism and Pharmacokinetics, Research and Early Development, Cardiovascular, Renal and Metabolism (CVRM), BioPharmaceuticals R&D, AstraZeneca, Pepparedsleden 1, SE-431 83 Mölndal, Sweden

[4] Vienna Doctoral School of Pharmaceutical, Nutritional and Sport Sciences (PhaNuSpo), University of Vienna, 1090 Vienna, Austria

[5] Dipartimento di Scienze Farmaceutiche, Università degli Studi di Milano, I-20133 Milano, Italy

* Corresponding authors: Filip Miljković, E-mail: filip.miljkovic@astrazeneca.com; Johannes Kirchmair, E-mail: johannes.kirchmair@univie.ac.at.

Table S1. Murcko scaffolds in the AZ Compound Set that are also represented in the MetaQSAR data set or the Approved Drugs set.

Murcko scaffold	SMILES	No. compounds in the AZ Compound set	No. compounds in the MetaQSAR data set	No. compounds in the Approved Drugs set
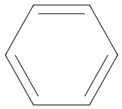	c1ccccc1	1	257	210
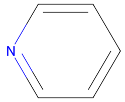	c1ccncc1	1	25	16
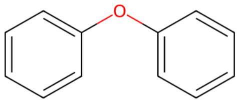	c1ccc(Oc2ccccc2)cc1	1	9	7
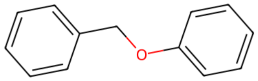	c1ccc(COc2ccccc2)cc1	1	7	4
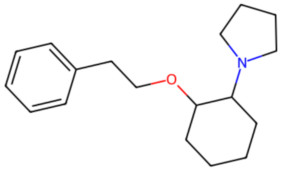	c1ccc(CCOC2CCCCC2N2CCCC2)cc1	1	0	1

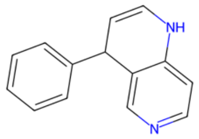	C1=CC(c2ccccc2)c2cnccc2N1	1	0	1
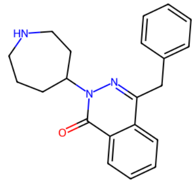	O=C(CCOCc1ccccc1)N1CCC2=NNC(=O)C2(Cc2ccccc2)C1	1	1	1
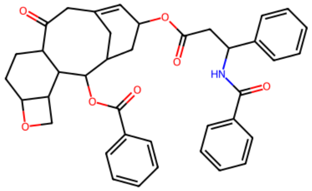	O=C(CC(NC(=O)c1ccccc1)c1ccccc1)OC1C=C2CC(=O)C3CCC4OCC4C3C(OC(=O)c3ccccc3)C(C2)C1	1	1	1

Table S2. Unique atom environments represented by the AZ SoM_exact data set and the MetaQSAR data set.

	AZ SoM_exact data set		MetaQSAR data set				
Rooted Morgan fingerprint radius	No. unique atom environments	Atom diversity[a]	No. unique atom environments	Atom diversity[a]	Overlap of unique atom environments	Overlap as % of AZ SoM_exact	Overlap as % of MetaQSAR
1	525	9.51	1951	19.95	461	87.81	23.63
2	1708	2.92	11425	3.41	797	46.66	6.98
3	2478	2.01	21075	1.85	433	17.47	2.05
4	2888	1.73	26816	1.45	228	7.89	0.85
5	3226	1.55	30430	1.28	137	4.25	0.45

[a] Average number of atoms with unique atom environment.

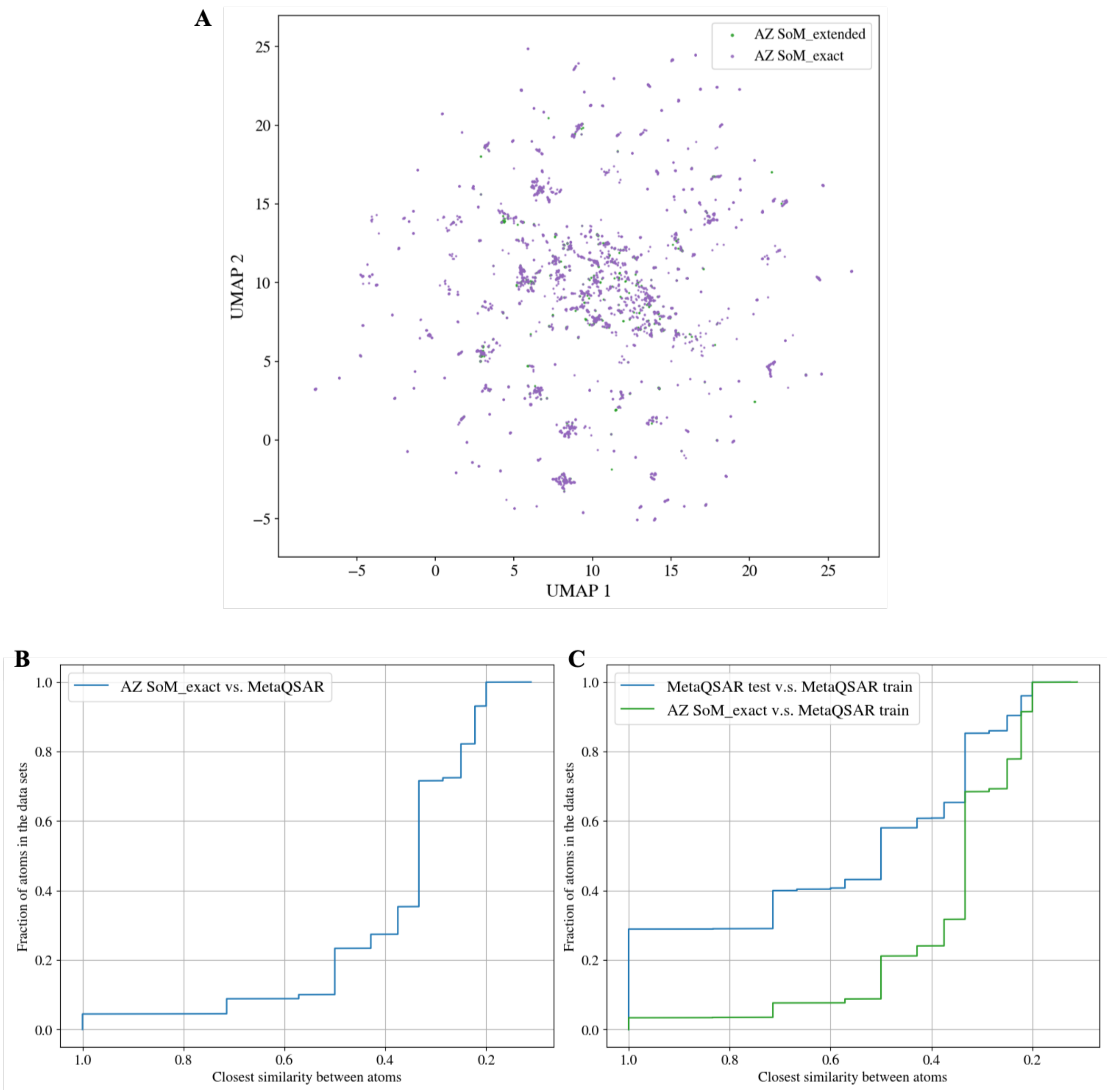

Figure S1. (A) UMAP plot of the atoms represented in the AZ SoM_extended and AZ SoM_exact data sets (UMAP generated from CDPKit FAME descriptors with a radius of 5). (B) Distribution of atom environment similarities between the atoms included in the AZ SoM_exact set and their nearest neighbors in the MetaQSAR data set (derived from rooted Morgan fingerprints with a radius of 5 and 2048 bits). (C) Distribution of atom environment similarities between the atoms included in the MetaQSAR test set and AZ SoM_exact set and their nearest neighbors in the MetaQSAR training set (derived from rooted Morgan fingerprints with a radius of 5 and 2048 bits).

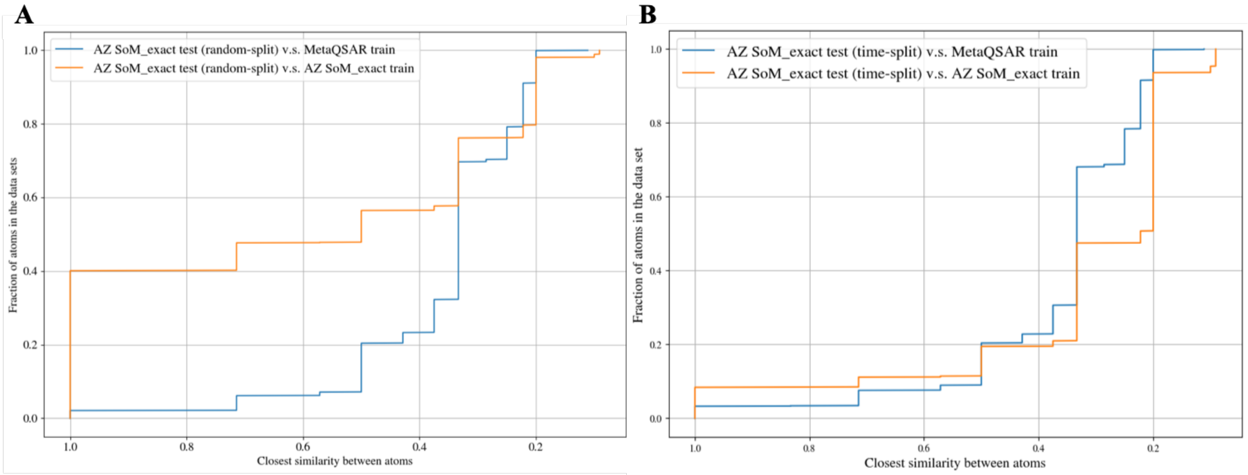

Figure S2. Distribution of atom environment similarities between the atoms included in the AZ SoM_exact test sets and their nearest neighbors in the MetaQSAR training set and AZ SoM_exact training sets, respectively (derived from rooted Morgan fingerprints with a radius of 5 and 2048 bits). A) for random-split AZ SoM_exact training set and test set, and B) for time-split.
